# Anogenital Human Papillomavirus Infection and HIV Infection Outcomes Among Peruvian Transgender Women: Results from a Cohort Study

**DOI:** 10.1089/trgh.2016.0001

**Published:** 2016-05-01

**Authors:** Brandon Brown, Jerome T. Galea, Gita Byraiah, Tonia Poteat, Segundo R. Leon, Gino Calvo, Hugo Sánchez, Thomas Coates, Jeffrey D. Klausner

**Affiliations:** ^1^ Center for Healthy Communities, SOM, University of California Riverside, Riverside, California.; ^2^ Socios en Salud, Lima, Peru.; ^3^ Epicentro Salud, Lima, Peru.; ^4^ Cooper Medical School of Rowan University, Camden, New Jersey.; ^5^ Johns Hopkins Bloomberg School of Public Health, Baltimore, Maryland.; ^6^ Medicine Infectious Diseases, University of California, Los Angeles, California.

**Keywords:** HIV/AIDS, HPV, sexually transmitted infections, transgender, anogenital warts

## Abstract

Latin American transgender women are highly vulnerable to HIV infection, and although much is known about factors associated with HIV infection in this population, little is known about the association of human papilloma virus (HPV) with HIV infection. We investigated anogenital HPV and cumulative HIV incidence among 68, initially HIV uninfected, Peruvian transgender women enrolled into a 2-year, prospective cohort study: 95.6% had at least one anogenital HPV genotype at baseline, 19.1% had visible anogenital warts, and 6.0% became infected with HIV over the course of the study. Due to the high anogenital HPV prevalence, this population would likely benefit from early immunization with the HPV vaccine.

## Background

Worldwide, transgender women are disproportionally affected by HIV, with an odds 49 times the general population.^1^ In Peru, HIV prevalence in transgender women is estimated at 30%.^2^ Although HIV incidence and human papilloma virus (HPV) acquisition have been increasingly studied in Peru,^2–4^ less is known about the risks of acquiring HIV when already infected with HPV and the role that visible anogenital warts may play.^5,6^ Specifically among transgender women, there may be a heightened risk for HPV infection and development of anogenital warts as a result of receptive anal intercourse.^7^ HPV infection is associated with the development of anogenital cancers, and coinfection with HIV accelerates the development of HPV-associated cancer.^8,9^

In a systematic review of the literature on HPV infection and acquisition of HIV infection across multiple populations, most studies found an association between HPV infection and subsequent infection with HIV, with nearly double the risk of acquiring HIV infection in those previously infected with HPV.^10^ The suggested mechanism of biological plausibility given for this association is epithelial disruption of the genital tract by HPV infection, which could facilitate HIV acquisition.^11^ Studies among men who have sex with men (MSM) have demonstrated an association between HPV-related anogenital warts and HIV acquisition.^5^ A study by Chin-Hong et al. found that the presence of two or more HPV genotypes in the anus was associated with HIV seroconversion.^6^ However, no studies have assessed the risks of incident HIV infection in transgender women based on anogenital HPV infection and the presence of anogenital warts. The study objectives were to (1) describe baseline prevalence for anogenital warts and anogenital HPV types in this population, (2) determine the incidence of anogenital HPV and HIV infection over the course of the study period, and (3) explore the association between baseline anogenital HPV types and incident HIV infection.

## Study Overview

We used data from a larger cohort study of 600 HIV uninfected Peruvian transgender women and MSM enrolled in Project VIVA, a longitudinal 2-year study described elsewhere.^12^ The study was designed to recruit both transgender women and MSM, with an expected enrollment of 5–20% transgender women based on the gender distribution at the recruitment venue. The relationship between baseline anogenital wart presence, anogenital HPV infection, and incident HIV infection in initially HIV uninfected transgender women was assessed.

## Methods

Using convenience sampling, participants were recruited from Epicentro Salud, a community health center providing care to MSM and transgender women in Lima, Peru. Study inclusion criteria were as follows: born anatomically male, aged 18–40 years, self-reported anal sex with a male within 12 months before study enrollment, resident of metropolitan Lima with no plans to move during the study period, and being HIV uninfected at enrollment. Participants were excluded if they had previously participated in an HIV or HPV vaccine trial. Details of baseline methodology were previously published.^7,12,13^ For the following analysis, only participants who identified as a gender different from male were included. The 2-year prospective clinical study consisted of twice-yearly surveys and specimen collection. The computerized survey was self-administered and addressed sexual and perisexual behaviors and practices, whereas the clinical examination included testing for HPV and HIV infection. The study was approved by institutional review boards at the University of California, Los Angeles (UCLA) and Impacta Salud y Educación in Peru.

### Specimen collection and testing

At the initial visit, participants were asked about history of anogenital warts. During the clinical examination, pre-moistened Dacron swabs were used to collect specimens from the penis coronal sulcus/glans, shaft, scrotum, and anus and then pooled into one sample per participant for a single HPV test result per person. That was done to increase cost-effectiveness, following a previously established protocol to define presence of anogenital infection.^14^ Samples were stored at −80°C before transfer to the testing laboratory. At the Moffitt Cancer Center Laboratory, DNA was extracted using the QIAamp Media MDx Kit (Qiagen), followed by PCR and HPV genotyping. Samples that tested positive for β-globin or at least one HPV genotype were considered adequate and were included in the analysis (overall β-globin positivity 98%). The Linear Array assay (Roche Diagnostics) was used for detection of 37 HPV genotypes classified as high risk (oncogenic; HPV 16, 18, 31, 33, 35, 39, 45, 51, 52, 56, 58, 59, and 68) or low risk (nononcogenic; HPV 6, 11, 26, 40, 42, 53, 54, 55, 61, 62, 64, 66, 67, 69, 70, 71, 72, 73, 81, 82, 82 subtype IS39, 83, 84, and 89 [formerly CP6108]).^15^ HIV infection status was ascertained using the Determine HIV-1/2 Combo Ag/Ab test (Alere, Inc.) and confirmed by indirect immunofluorescence assay (in-house test, Peruvian National Institute of Health); individuals testing positive were linked to the national HIV program for free care.

### Data analysis

Univariate descriptive statistics were used to examine baseline variables, prevalence of HPV infection and anogenital warts, and incident HIV infection. Chi-square tests were conducted to assess the association of anogenital warts with incident HIV. All analyses were conducted using Stata Version 12.0 (Stata Corp.).

## Results

Of 68 transgender women, the mean age was 25.2 years (range 18–39), with 79.4% completing secondary school and 20.4% completing university education. Over half (52.9%) had a current partner and 42.7% had anal sex for the first time before 14 years of age. Sex partners of transgender women were mostly men (95.1%), but some (4.5%) transgender women reported sex with men and women. During anal sex with men, the majority of participants were exclusively (34.4%) or mostly (52.5%) receptive, with 6.6% equally receptive and insertive. Transgender women had a mean frequency of 72 episodes of anal intercourse during the previous 6 months, with 87.5% reporting condom use during their last anal sex encounter. The majority of participants (82.1%) received money or favors for sex in the past 6 months. Nearly half of participants (42.2%) reported sex under the influence of alcohol in the past month. Use of rectal douches in relation to receptive anal intercourse was reported by nearly half (47%) of transgender women. Approximately one-third (36.9%) of participants had heard of HPV before participating in this study.

**FIG. 1.**
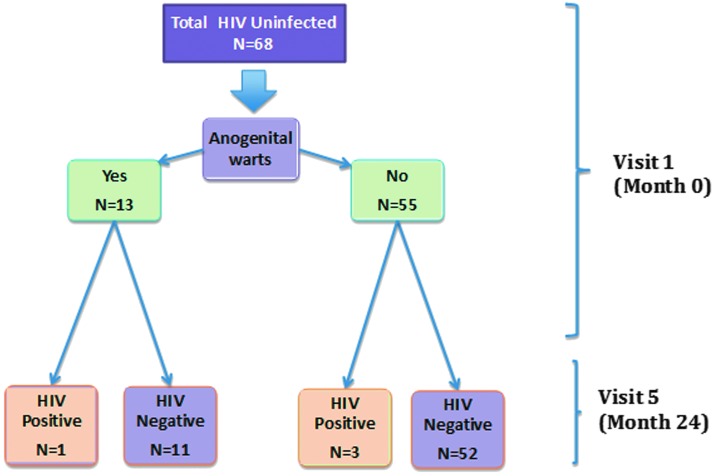
Study diagram of Peruvian transgender women enrolled in the VIVA Study in Lima, Peru (one person missing from final follow-up).

**FIG. 2.**
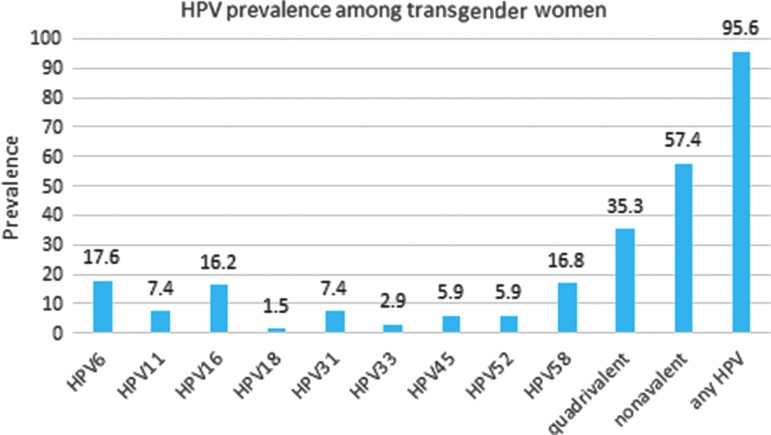
Prevalence of individual HPV genotypes, those in quadrivalent HPV vaccine, those in nonavalent HPV vaccine, and any HPV genotype (37 total types) among 68 Peruvian transgender women. HPV, human papilloma virus.

Anogenital warts were present in 19.1% of transgender women at the baseline visit, with the majority of those having anal warts only (Fig. 1). The prevalence of any anogenital HPV infection was 95.6%. Vaccine-related genotypes included HPV6 (17.6%), HPV11 (7.4%), HPV16 (16.2%), HPV18 (1.5%), HPV31 (7.4%), HPV33 (2.9%), HPV45 (5.9%), HPV52 (5.9%), and HPV58 (16.8%). Transgender women had an 82.3% prevalence of LRHPV (HPV types 6 and 11), 58.8% HRHPV (HPV types 16, 18, 31, 33, 45, 52, and 58), and 66.2% had more than one HPV type (Fig. 2). Of those who reported condom use during their last episode of anal intercourse, 84.6% had the presence of any HPV subtype. HIV was acquired by 6.0% (*n*=4) of transgender women across the 2-year study period. Of the four transgender women who tested HIV positive, one had anogenital warts (*p*=0.55).

## Conclusions

Anogenital warts were common among transgender women in this sample and the overwhelming majority had prevalent anogenital HPV infection, despite reports of condom use at last anal sex. Consistent condom use has been shown to increase anogenital HPV infection clearance rates in other populations by preventing reinfection.^16^ The high rates of HPV infection among transgender women in our study may have been related to less than 100% condom use, the frequency of anal intercourse, and potential exposure to a variety of HPV genotypes while engaged in sex work. Although a high percentage of transgender women in the study were infected with more than one HPV genotype, being infected with multiple genotypes was not associated with HIV seroconversion as found in a previous study.^6^

An overall annual HIV incidence rate of 3% among transgender women in Peru is very high and consistent with the heavy burden of HIV among transgender women.^2^ Although reported condom use for anal sex at the last sexual encounter was high and higher than in previous studies with transgender women in Lima (87.5% vs. 75.1%), condom promotion alone has not been sufficient to curb the HIV epidemic in this population.^2^ More than half of the transgender women in the study were infected with high-risk HPV types that are targeted by the nonavalent HPV vaccine. Thus, those women could have benefited from immunization before exposure. New HIV infections and high prevalent anogential HPV in the setting of higher than typical condom use highlight the need for additional prevention measures, such as the HPV vaccine and HIV pre-exposure prophylaxis.

Education will also be key to reduce HPV transmission. A minority of transgender women had heard of HPV infection before participating in this study. Previously reported data from this cohort demonstrated that transgender women were four times as likely as MSM to believe that HPV infection had been resolved when there were no longer visible genital warts.^13^ Identifying and addressing those knowledge gaps are essential components of HIV and sexually transmitted infection risk reduction strategies.

Our study was limited by the use of visual inspection for the ascertainment of anogenital warts. Although costprohibitive in this study, biopsy is the gold standard for diagnosis of HPV-related warts and would have been able to confirm the lesions. In addition, since we pooled samples from the penis coronal sulcus/glans, shaft, scrotum, and anus, we could not identify the exact anatomical site of HPV infection. Due the use of convenience sampling, our findings may not be generalizable to beyond the study population. The small sample size of transgender women reduced the power of the study to detect significant relationships that may have been present.

We report on prevalence of anogenital HPV infection and anogenital warts among transgender women, and to our knowledge we are the first to test an association between anogenital warts and HIV incidence among transgender women. Future studies should explore hypothesized relationships between HIV infection and the location of HPV infection in this population compared with MSM and natal female sex workers, and control for other potential confounders including use of gender-affirming hormones, douching, and longer term condom use. Larger sample sizes and the use of biopsy confirmation for anogenital warts would also strengthen future studies.

## Abbreviation Used

HPV: human papilloma virus
